# Lactate dehydrogenase in *Toxoplasma gondii* controls virulence, bradyzoite differentiation, and chronic infection

**DOI:** 10.1371/journal.pone.0173745

**Published:** 2017-03-21

**Authors:** Abdelbaset E. Abdelbaset, Barbara A. Fox, Mohamed H. Karram, Mahmoud R. Abd Ellah, David J. Bzik, Makoto Igarashi

**Affiliations:** 1 National Research Center for Protozoan Diseases, Obihiro University of Agriculture and Veterinary Medicine, Hokkaido, Japan; 2 Clinical laboratory diagnosis, Department of Animal medicine, Faculty of veterinary medicine, Assiut University, Assiut, Egypt; 3 Department of Microbiology & Immunology, The Geisel School of Medicine at Dartmouth, Lebanon, New Hampshire, United States of America; Centre National de la Recherche Scientifique, FRANCE

## Abstract

In the asexual stages, *Toxoplasma gondii* stage converts between acute phase rapidly replicating tachyzoites and chronic phase slowly dividing bradyzoites. Correspondingly, *T*. *gondii* differentially expresses two distinct genes and isoforms of the lactate dehydrogenase enzyme, expressing LDH1 exclusively in the tachyzoite stage and LDH2 preferentially in the bradyzoite stage. LDH catalyzes the interconversion of pyruvate and lactate in anaerobic growth conditions and is utilized for energy supply, however, the precise role of LDH1 and LDH2 in parasite biology in the asexual stages is still unclear. Here, we investigated the biological role of LDH1 and LDH2 in the asexual stages, and the vaccine strain potential of deletion mutants lacking LDH1, LDH2, or both genes (*Δldh1*, *Δldh2* and *Δldh1/2*). Deletion of LDH1 reduced acute parasite virulence, impaired bradyzoite differentiation *in vitro*, and markedly reduced chronic stage cyst burdens *in vivo*. In contrast, deletion of LDH2 impaired chronic stage cyst burdens without affecting virulence or bradyzoite differentiation. Deletion of both LDH1 and LDH2 induced a more severe defect in chronic stage cyst burdens. These LDH mutant phenotypes were not associated with any growth defect. Vaccination of mice with a low dose of mutants deleted for LDH elicited effective protective immunity to lethal challenge infection, demonstrating the vaccine potential of LDH deletion mutants. These results suggest that lactate dehydrogenase in *T*. *gondii* controls virulence, bradyzoite differentiation, and chronic infection and reveals the potential of LDH mutants as vaccine strains.

## Introduction

*Toxoplasma gondii* is an obligate intracellular parasite belonging to Apicomplexa. This parasite has two asexual stages, namely tachyzoite and bradyzoite stages. Tachyzoites contribute to acute infection to disseminate parasites to the brain and other tissues. The tachyzoite to the bradyzoite stage conversion can be *in vitro* induced by alkaline pH 8.0–8.2, high temperature (43°C), interferon (IFN)-γ, or mitochondrial inhibitors [1–4]. Additionally, host immunological attack of the acute infection triggers stage conversion [5], however, the molecular mechanisms of stage conversion are still largely unknown. On the other hand, the bradyzoite stage is involved in the chronic stage. Bradyzoites grow slowly, form tissue cysts, and persist for the lifetime of the host. Host immune deficiency can lead to stage conversion of bradyzoites back to tachyzoites, and reactivated infection.

Many bradyzoite specific/upregulated genes have been identified [6–16]. For example, expression of *LDH2* is strongly upregulated during bradyzoite differentiation [17]. *T*. *gondii* has two lactate dehydrogenase (LDH) isoforms, LDH1 and LDH2, encoded by distinct genes (*LDH1* and *LDH2*). These enzymes catalyze the interconversion of pyruvate and lactate and are utilized for energy supply in anaerobic growth condition [18]. In tachyzoite stages, LDH2 expression is not detectable. In contrast, LDH1 expression is abundant in the tachyzoite stages, and appears to play a major role of pyruvate-lactate catalysis in this stage. During bradyzoite differentiation, LDH1 expression is suppressed post-transcriptionally/translationally and LDH2 expression is induced transcriptionally [19]. Both enzymes show nearly identical catalytic activities without any significant differences in enzyme kinetics. Compounds that inhibit LDH activity have been shown to inhibit growth of tachyzoites in cultured fibroblasts [20]. In addition, parasites with engineered knockdown of the expression of LDH1 or LDH2 exhibited slower growth rates *in vitro*, reduced virulence, and impaired development of cyst burdens in *vivo* [21]. In this study, we developed precisely targeted LDH knockout strains by deleting *LDH1*, *LDH2*, or *LDH1* and *LDH2* to further investigate the role of lactate dehydrogenase during the tachyzoite and the bradyzoite stages, and the vaccine potential of LDH deletion mutants.

## Materials and methods

### Ethics statement

All mouse work was performed in strict accordance with the recommendations in the Guide for the Care and Use of Laboratory Animals of Obihiro University of Agriculture and Veterinary Medicine. The protocol was approved by the Committee on the Ethics of Animal Experiments of Obihiro University of Agriculture and Veterinary Medicine (Permit Number: 27–134). Health condition was monitored daily. Mice with weight loss of 20% or more in 5 days were considered lethal and euthanasia was performed under isoflurane anesthesia.

### Parasites

The weakly virulent type II Pru strain with hypoxanthine-xanthine-guanine phosphoribosyl transferase (HXGPRT) and *Ku80* knockouts of *T*. *gondii* (PruΔ Ku80::HXGPRT) was used [22]. *T*. *gondii* tachyzoites were maintained in our laboratory through serial passage in Vero or human foreskin fibroblast (HFF) cells grown in modified Eagle’s medium (Sigma-Aldrich, Dorset, UK) containing 5% fetal calf serum (FCS) at 37°C with 5% CO_2_.

### *In vitro* bradyzoites differentiation

*In vitro* bradyzoites differentiation was conducted by alkaline treatment of confluent HFF cells. Confluent HFF cells were infected with tachyzoites and differentiation was induced by culture in sodium bicarbonate-free RPMI1640 containing 25 mM HEPES [pH 8.1] and 1% FCS three hours after infection and incubated at 37°C without CO_2_.

### Preparation of tachyzoite and bradyzoite lysates

Preparation of tachyzoite lysates were performed as previously described [23]. The infected HFF monolayers were washed with cold phosphate-buffered saline (PBS) and scraped. Cell pellets were resuspended in medium, passed through a 27-gauge needle and filtered through a 5.0 μm-pore filter (Millipore, Bedford, MA, U.S.A.). After centrifugation at 2,000 × g for 5 min at 4°C, the pellet was resuspended with RIPA buffer (50 mM Tris-HCl, pH 7.5, 150 mM NaCl, 1 mM EDTA, 0.25 mM sodium deoxycholate, 0.1% Triton X-100 and 1% Nonidet P-40). The tachyzoite lysates were recovered after centrifugation at 2,000 × g for 5 min.

Preparation of bradyzoite lysates was done as described previously [24, 25]. Confluent HFF cells were infected with tachyzoites and differentiation was *in vitro* induced by culture in RPMI media [pH 8.1]. Three days after induction, the infected HFF monolayers were washed with cold phosphate-buffered saline (PBS) and scraped. Cell pellets were resuspended in medium and passed through a 27-gauge needle to disrupt host cells. Host cell debris was removed by using Arabic density gradient centrifugation (Sigma). 2 ml of gum Arabic of 1.07 specific gravity (sg) was added to each tube and 2 ml of gum Arabic of 1.05 sg was thereafter slowly dispensed. Five milliliters of collected bradyzoites suspension samples were also overlaid slowly to each tube. After 10 min of centrifugation at 2,100×g at 15°C, the parasite pellets containing cysts, freed bradyzoites and uninduced tachyzoites from all the tubes were added together in one tube. The collected parasites from the procedure mentioned above were washed twice with 10 ml of 1 × PBS by centrifugation at 2,100×g at 4°C for 15 min and lysed by three freeze–thaw cycles in RIPA buffer. The lysates were recovered after centrifugation at 2,000 × g for 5 min.

### *LDH1* and *LDH2* cDNA cloning and sequencing analysis

*T*. *gondii LDH1* (TGME49_232350) and *LDH2* (TGME49_291040) cDNA containing entire coding regions was RT-PCR amplified from ME49 strain total RNA as a template using primer 1 and 2 (LDH1) and primer 3 and 4 (LDH2), respectively as described in Table 1. The amplified cDNA was double digested with *Bam*HI and *Not*I and subcloned into identical restriction sites of bacterial expression plasmid with GST-tag, pGEX-6P-2 (GE Healthcare, Buckinghamshire, UK) and mammalian expression plasmid with V5-tag, pcDNA6/V5-His C (Invitrogen, CA, USA). Plasmids were transformed into *Escherichia coli* DH5α-competent cells. Individual colonies were grown at 37°C overnight with rotary shaking in 1 ml of Luria-Bertani (LB) medium with 50 μg/ml of ampicillin. Plasmid DNA was extracted by alkali lysis and ethanol precipitation [26]. After plasmid preparation, 1 μl (out of 10 μl) of each sample was treated with the restriction enzymes, *Bam*HI and *Not*I, to check for the presence of insert DNA. Only plasmids containing the expected insert size were used for the subsequent nucleotide sequencing analysis.

**Table 1:** Oligonucleotides used in this study. The sequences corresponding to the recognition sites of restriction endonucleases are underlined.

1	LDH1-ORF forward	TTTTGGATCCATGGCACCCGCACTTGTGCAGAGG
2	LDH1-ORF reverse	TTTTGCGGCCGCCGCCTGAAGAGCAGCAACCGCCTT
3	LDH2-ORF forward	TTTTGGATCCATGACGGGTACCGTTAGCAGAAGA
4	LDH2-ORF reverse	TTTTGCGGCCGCACCCAGCGCCGCTAAACTCTTATT
5	LDH1-5' forward	TTTTGCGGCCGCGAGAACGGGGAAACAGGAGAAAAG
6	LDH1-5' reverse	TTTTAGATCTTTTGCTGACTAAAAATGAGCGGGA
7	LDH1-3' forward	TTTTATCGATATGATTTATACACGCGTTTGCAAC
8	LDH1-3' reverse	TTTTGGGCCCCGAAGAACGAATACGCTCTTTTCCG
9	LDH2-5' forward	TTTTTCTAGAGTTTCCATGCAGACGCGGAGTTTG
10	LDH2-5' reverse	TTTTAGATCTGGTGGAAGTGAAGTACGAATGCCG
11	LDH2-3' forward	TTTTATCGATACAAGTACGCAGGCGGGCAGCACA
12	LDH2-3' reverse	TTTTGGGCCCCATCCGGACCCACGATTGCTAGCG
13	LDH1-5' outside	GGCCGACGAAGGGAGACCGGAATGAGG
14	LDH1-3' outside	GTACCATCAGGGCTATAGTTCCGAGGG
15	LDH2-5' outside	CTACTTCAGATGGAACCGAATAGCAAT
16	LDH2-3' outside	CGCCCGAAGAGAGTTATGGCAGGGATC
17	DHFR inside reverse	CGCGGGGGGTGAAAATCGAATGACATG
18	DHFR inside forward	CTTTCCTTCTATGCACTTGCAGGAT
19	HXGPRT inside forward	ATATGACCGTCTGGCAAACATGGCT
20	HXGPRT inside reverse	AATCAACCGAATTCATTTGGCGAGA

Cycle sequencing reactions were carried out using a BigDye Terminator Cycle Sequencing kit Ver. 3.1 according to the manufacturer’s protocol (Applied Biosystems, CA, USA), and each sample was analyzed using an ABI PRISM 3100 Genetic Analyzer (Applied Biosystems, CA, USA). The nucleotide sequence of the plasmid was compared with that of the *Toxoplasma* genome sequence in ToxoDB (http://toxodb.org/toxo/).

### Production of recombinant LDH2 protein

The pGEX-6P-2 plasmids with *LDH2* cDNA were transformed into *E*. *coli* DH5α-competent cells and grown in LB medium supplemented with 50 μg/ml ampicillin with vigorous shaking at 37°C up to an optical density of 0.6 measured at 600 nm. Then, the GST-fused protein was induced with isopropyl-β-D-thiogalactopyranoside (IPTG) to a final concentration of 1 mM with mild shaking at 25°C overnight. The cells were centrifuged and the bacterial pellet was resuspended in phosphate buffered saline (PBS) containing 10μg/ml of lysozyme, and then stored at -20°C. After thawing, the cells were disrupted by sonication on ice, cell debris was removed by centrifugation (3,500 rpm, 20 min), and the resulting supernatant was recovered to a new tube. Recombinant proteins in the soluble fraction were affinity purified by glutathione-Sepharose beads according to the manufacturer’s protocols (GE Healthcare, Buckinghamshire, UK). Beads were washed three times with PBS and then bound proteins were eluted with elution buffer (200 mM NaCl, 20 mM reduced glutathione, 100 mM Tris-HCl [pH 9.5] and 5 mM EDTA). The eluted fractions were dialyzed against PBS and the amount of recombinant proteins were calculated using the Coomassie protein assay reagent kit according to the manufacturer’s protocols (Thermo Scientific, MA, USA).

### Production of polyclonal anti- LDH antiserum

The polyclonal anti-LDH2 antisera were produced in Balb/c mice. Mice were immunized as described previously [24]. In brief, mice were immunized subcutaneously at three different inoculation sites with 10 μg of recombinant LDH2 protein emulsified with an equal volume of Freund’s complete adjuvant. Two weeks later, mice were immunized with the same dose of antigen emulsified with Freund’s incomplete adjuvant. On day 28, mice were immunized with one more dose of antigen with Freund’s incomplete adjuvant. Mice were sacrificed 7 days after last immunization under isoflurane anesthesia and blood for serum preparation was collected. All efforts were made to minimize suffering. The cross-reactivity of the sera collected was tested and confirmed by western blot and indirect immunofluorescence assay (IFA).

### Western blot analysis

Samples were dissolved in SDS-PAGE sample buffer (62.5 mM Tris-HCl [pH 6.8], 2% (w/v) SDS, 140 mM 2-mercaptoethanol, 10% (w/v) glycerol and 0.02% (w/v) bromophenol blue), heated at 98°C for 5 min and separated on a 10 or 12% polyacrylamide gel. All separated proteins were electrically transferred onto a polyvinylidene fluoride (PVDF) membrane (Immobilon-P, Millipore, MA, USA) using a Western blot apparatus (HorizeBlot Type AE-6677, ATTO Bioscience & Biotechnology, Tokyo, Japan). The membrane was blocked with PBS containing 1% (w/v) skimmed milk (PBS-M) for 1 hour, and then incubated with primary antibodies at 1:500 in PBS-M for 1 hour. After washing three times with PBS containing 0.05% Tween 20 (PBS-T), the membrane was incubated with anti-mouse immunoglobulin G (IgG) conjugated with horseradish peroxidase from sheep (GE Healthcare, Buckinghamshire, UK) diluted at 1:1000 in PBS-M for 1 hour. For detection of *T*. *gondii* actin (ACT1) protein, anti-rabbit immunoglobulin G (IgG) conjugated with horseradish peroxidase from sheep (GE Healthcare, Buckinghamshire, UK) was used. After washing three times, specific protein was detected by detection solution (0.1 M Tris, pH 8.5, 1.25 mM luminol, 0.2 mM coumaric acid, 0.075% H_2_O_2_) and exposed to X ray film [24]. Molecular mass standards (SeeBlue plus2 Pre-stained standard–Life Technologies, CA, USA) were used for size determination.

### Indirect immunofluorescence assay

The indirect immunofluorescence assay (IFA) was performed as described previously [25]. Parasites were inoculated onto glass coverslips with confluent monolayers of HFF cells and incubated at 37°C. After washing with PBS three times, the coverslips were fixed and permeabilized with 4% formaldehyde-0.2% Triton X-100 in PBS, pH 7.0, for 15 min. After washing with PBS three times, samples were blocked with blocking solution (3% bovine serum albumin (BSA) fraction V in PBS, pH 7.0) for 20 min. Samples were then incubated for 1 hour with primary antibodies diluted 1:500 in blocking solution. Anti- LDH1/2, IMC1 and SAG1 antibodies [16] were used. After washing three times with PBS, samples were incubated for 1 hour with goat anti-mouse or anti-rabbit secondary antibody conjugated with Alexa fluor 488 or 594 diluted 1:1000 in blocking solution. For cyst wall staining, FITC-conjugated *Dolichos biflorus* lectin (Vector laboratories Inc., CA, USA) was used. After washing three times with PBS and once with water, samples were mounted with Mowiol (9.6% Mowiol, 24% glycerol, 100 mM Tris [pH 8.5]) and examined using a Leica TCS NT Confocal Laser Scanning Microscope (Leica Microsystems, Wetzlar, Germany).

### Deletion of the gene encoding *LDH1* and *LDH2*

For generation of a *LDH1* and *LDH2* deletion plasmids, upstream (3.2 or 3.0 kbp, respectively) and downstream (3.2 or 3.4 kbp, respectively) flanking regions of the *LDH1* and *LDH2* loci were amplified from *T*. *gondii* strain ME49 genomic DNA using primers in Table 1 and subcloned into pBluescript SK II vector. The HXGPRT or DHFR-TS expression cassette was subcloned between them. The GFP expression cassette was subcloned outside of deletion constructs for negative selection. One hundred μg of the final constructs, p*LDH1*::HXGPRT and p*LDH2*::DHFR, were transfected by electroporation into *T*. *gondii* strain PruΔKu80::hxgprt [27]. For selection of transformants, the transfected parasites were grown in medium containing 25μg/ml mycophenolic acid and 50μg/ml xanthine for LDH1 knockout and 1μM pyrimethamine for LDH2 knockout. After 15 days of selection, the parasites were cloned by limiting dilution. GFP-negative parasites were isolated and the absence of the *LDH1* and *LDH2* genes were screened by PCR for the coding regions. Homologous recombination was examined by PCR using primers of the *LDH1* and *LDH2* flanking region and inside of the HXGPRT or DHFR-TS expression cassette. Gene disruption was confirmed by IFA and western blot analysis using anti-LDH1 and anti-LDH2 polyclonal antisera.

### Analysis of the parasite growth kinetics

Parasites (1.0 × 10^6^ parasites per 100 mm tissue culture dish) were inoculated onto glass coverslips with confluent monolayers of HFF cells, and incubated in a 37°C CO_2_ incubator. The parasites were fixed and permeabilized (4% formaldehyde-0.2% Triton X-100 in PBS, pH 7.0, for 15 min) at indicated time points. Then the parasites were stained using anti-IMC1 polyclonal antisera (1:1000) [28] and the number of parasites per PV was counted. Each time point represents the mean value of about 100 PV of 5 different fields with a standard deviation. Data values were statistically analyzed using the Student’s *t*-test. *P*-values < 0.05 were considered to be statistically significant. Forty eight hours after infection, size of PV was analyzed by Image J software (https://imagej.nih.gov/ij/) instead of counting of parasites.

### Plaque assay

Parasites (1.0 × 10^4^ parasites per 100 mm tissue culture dish) were inoculated onto glass coverslips with confluent monolayers of HFF cells, and incubated in a 37°C CO_2_ incubator for 7 days. Then the parasites were fixed, permeabilized (4% formaldehyde–0.2% Triton X-100 in PBS, pH 7.0) for 15 min, stained using anti-IMC1 polyclonal antisera (1:1000) [28] and the long axis of plaque was measured. The plaque size represents the mean value of about 30 plaques with a standard deviation.

### Experimental infection to mice

The parasite virulence was examined by injecting indicated number of purified parasites intraperitoneally into 8-weeks old female Balb/c mice (n = 10). Survival rate was monitored every day for one month. Cyst number formed in brain of survived mice was examined. Brains were collected, 100μl from total 1 ml of homogenate was observed microscopically. Total number of cyst was calculated as multiplied by 10. To evaluate the protective immunity after primary infection, purified RH strain parasites (1.0 × 10^2^ parasites) were challenged intraperitoneally into randomly selected 5 mice of each group of 1.0 × 10^3^ parasites infection one month after primary infection. Uninfected mice were also challenged and used for naive control group. Survival rate was monitored every day for one month.

### Animals

Eight weeks old female Balb/c mice used in our experiments were purchased from CLEA, Japan. The mice were used for polyclonal antibody production and for the parasite virulence assay.

## Results

### Targeted disruption of genes encoding LDH1 and LDH2

**Fig 1:**
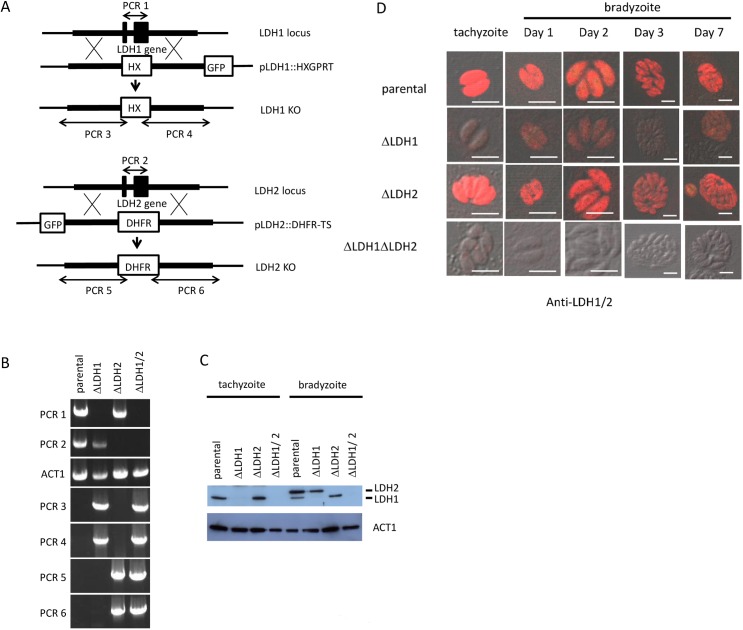
Construction of knockout strains. (A) Schematic diagram of LDH gene disruption strategy by double crossover homologous recombination in type II PruΔku80Δhxgprt using a plasmid, pΔLDH containing the 5’ and 3’ flanking region. HXGPRT and DHFR-TS selectable markers were used for selection. (B) PCR analysis for validation of gene knockouts. Signal of coding region of LDH1 (PCR1) is absent in *Δldh1* and *Δldh1Δldh2* strains. Signal of coding region of LDH2 (PCR2) is absent in *Δldh2* and *Δldh1Δldh2* strains. The actin coding region (ACT1) was used as a PCR control. Successful homologous recombination is indicated by the presence of signal of either LDH1 (PCR3 and 4) in *Δldh1* and *Δldh1/2* or LDH2 (PCR5 and 6) in *Δldh2* and *Δldh1/2* strains. (C) Western blot analysis of parental and knockout parasite lysates of tachyzoite and bradyzoite stage. Anti-LDH1/2 antibody which recognizes both LDH1 and 2 proteins was used. LDH1 signal is absent in *Δldh1* and *Δldh1/2* strains and LDH2 signal is absent in *Δldh2* and *Δldh1/2* strains. Although the predicted molecular weight of LDH1 and 2 is nearly identical (35.5 and 35.3 kDa, respectively), western blot antibody reactive bands of LDH2 migrated at an apparent higher molecular weight than LDH1. (D) Immunofluorescence staining of parental and knockout parasite lysates of tachyzoite and bradyzoite stage. Anti-LDH1/2 antibody which recognize both LDH1 and 2 proteins are used. In the *LDH1* disruption, weak signal gradually appeared after bradyzoite induction. No signal was detected in the candidate *LDH1/LDH2* double knockout parasites. White bar in photos indicates 10μm.

To better understand the role of LDH1 and LDH2, we generated knockout parasites by homologous recombination in PruΔKu80::hxgprt parental strain parasites. HXGPRT and DHFR-TS were used for LDH1 and LDH2 gene disruptions and selections, respectively (Fig 1A). In order to remove LDH activity completely, double knockout parasites were also generated using GFP for negative selection (Fig 1A). The GFP-negative parasites were then screened by PCR for the existence of *LDH1* (1.5 kbp) and *LDH2* (1.6 kbp) coding regions (Fig 1B, PCR 1 and PCR 2, respectively). PCR analysis showed that the parental strain had amplified bands for LDH1 and LDH2, however, these bands were absent in candidate knockout clones of the *LDH1* and *LDH2* deleted parasites (Fig 1B). *T*. *gondii* actin gene (ACT1) was also amplified as internal control. To confirm the successful homologous recombination, we performed PCR analysis using primers which annealed the *LDH1* flanking region outside of the disruption plasmid and HXGPRT expression cassette (Fig 1A, PCR 3 and PCR 4) or *LDH2* flanking region outside of the disruption plasmid and DHFR-TS expression cassette (Fig 1A, PCR 5 and PCR 6). These primers were designed to amplify the DNA fragment only in knockout parasites. PCR analysis showed that the candidate disruption parasites had amplified bands, and as expected, these PCR products were absent in the parental strain (Fig 1B).

We confirmed gene disruptions by western blot analysis (Fig 1C) using anti-LDH1/2 antibody. LDH1 protein was detected in the parental and the *LDH2* disruption parasite but was undetected in the *LDH1* disruption and *LDH1/LDH2* double knockout parasites in tachyzoite lysate. In bradyzoite lysates, the parental strain showed high intensity of LDH2 (upper band) and weak signal of LDH1 (lower band). The signal of LDH1 was absent in the candidate *LDH1* disruption parasite and the signal of LDH2 was absent in the candidate *LDH2* disruption parasite. The candidate *LDH1/LDH2* double knockout parasites lost both bands. Loading of samples was confirmed by anti-ACT1 antibody.

Immunofluorescence staining of the LDH knockout strains was also performed (Fig 1D). In parental and the candidate *LDH2* disruption parasite, signal was detected in tachyzoite and bradyzoite stages. In the *LDH1* disruption, weak signal gradually appeared after bradyzoite induction. No signal was detected in the candidate *LDH1/LDH2* double knockout parasites. These data indicate the successful generation of the *LDH1*, *LDH2*, and *LDH1/LDH2* knockout strains (*Δldh1*, *Δldh2* and *Δldh1Δldh2*).

### Growth kinetics of LDH knockout strains in the tachyzoite stage

**Fig 2:**
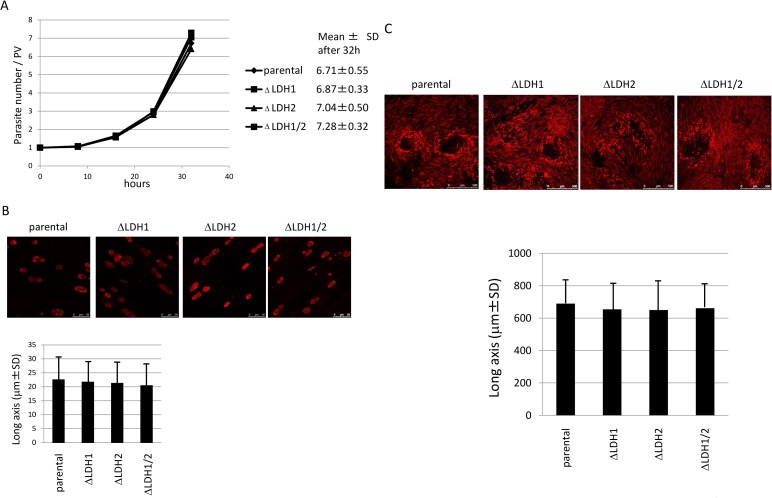
Growth kinetics. (A) The graph shows the average number of parasites per PV at the indicated time points. The numbers on the right side represent the average number of parasites per vacuole 32 hours after incubation. (B) Comparison of PV size with parental and knockout strains. PVs are visualized by immunostaining with anti-IMC antibodies (upper panel). Size of PVs was measured and represented as a graph (lower panel). (C) Parasite plaques were stained with anti-IMC1 antibody. The photos show the parental and knockout strains (upper panel). Plaque size was measured and represented as a graph (lower panel).

In order to examine whether LDH enzymes were required for tachyzoite growth *in vitro*, we performed growth assays of the knockout parasites. HFF cells were infected with the parental or knockout strain(s) and parasite numbers per parasitophorous vacuole (PV) were counted at indicated time points. As shown in Fig 2A, all four strains demonstrated similar growth rate. The result was confirmed by comparison of PV size 48 hours after infection (Fig 2B). Long axis of PV was measured and compared. PV size was essentially identical between parental and knockout strains. To further confirm this phenotype of the knockout parasites, we observed parasite plaque size 7 days after infection. Long axis of plaques was measured and compared. The plaque size of parental and knockout strains was not significantly different (Fig 2C).

### LDH1 deletion affects bradyzoite differentiation efficiency

**Fig 3:**
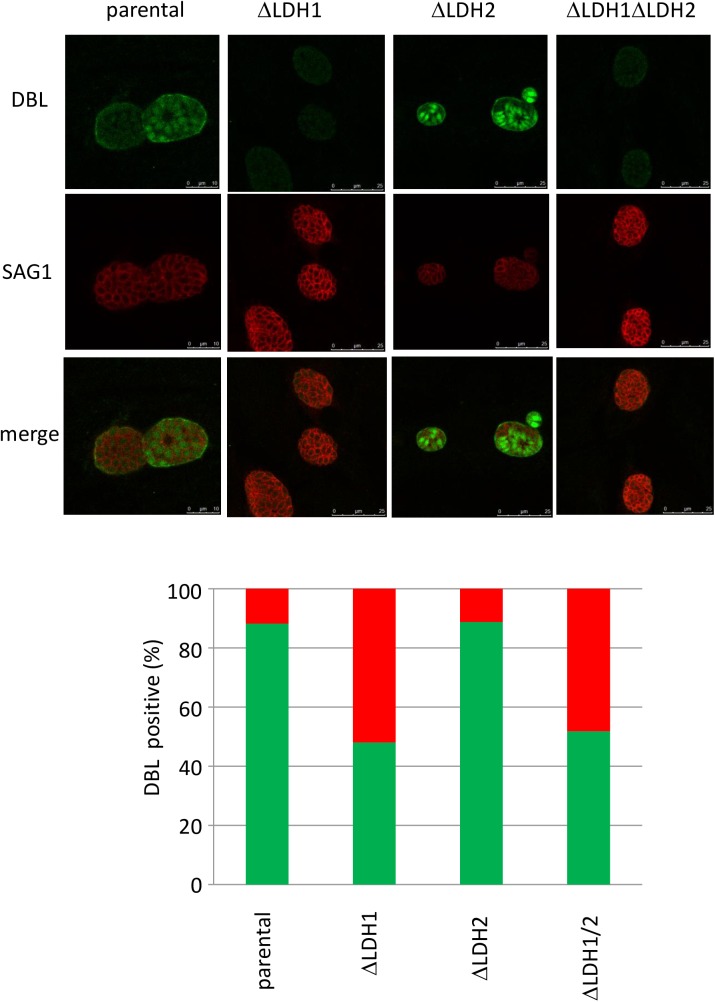
Analysis of stage conversion in parental and LDH knockout strains. *Dolichos biflorus* lectin (DBL) (green) was used for cyst wall staining and anti-SAG1 (red) antibody was used for tachyzoite marker (upper panel). Percentage of DBL positive parasites of each 100 PV from two independent assays was calculated (lower panel).

To examine the effect of LDH deletion on stage conversion *in vitro*, immunofluorescence staining of cyst wall by *Dolichos biflorus* lectin (DBL) and tachyzoite specific antigens SAG1 was carried out (Fig 3). Three days after bradyzoite induction, about 90% of parental as well as *Δldh2* strain vacuoles were positive for DBL straining. In contrast, only 50% of the *Δldh1* and *Δldh1Δldh2* strain vacuoles were positive for DBL (Fig 3 lower panel). In *Δldh1* and *Δldh1Δldh2* strain vacuoles that were negative for DBL staining, expression of tachyzoite stage specific SAG1 was high (Fig 3, upper panels). In contrast, in parental as well as *Δldh2* strain vacuoles that were positive for DBL staining, SAG1 expression was markedly reduced (Fig 3, upper panels). These results suggest that deletion of *LDH1* markedly impaired bradyzoite differentiation, while the deletion of *LDH2* did not impair bradyzoite differention.

### Infection study of LDH knockout

**Fig 4:**
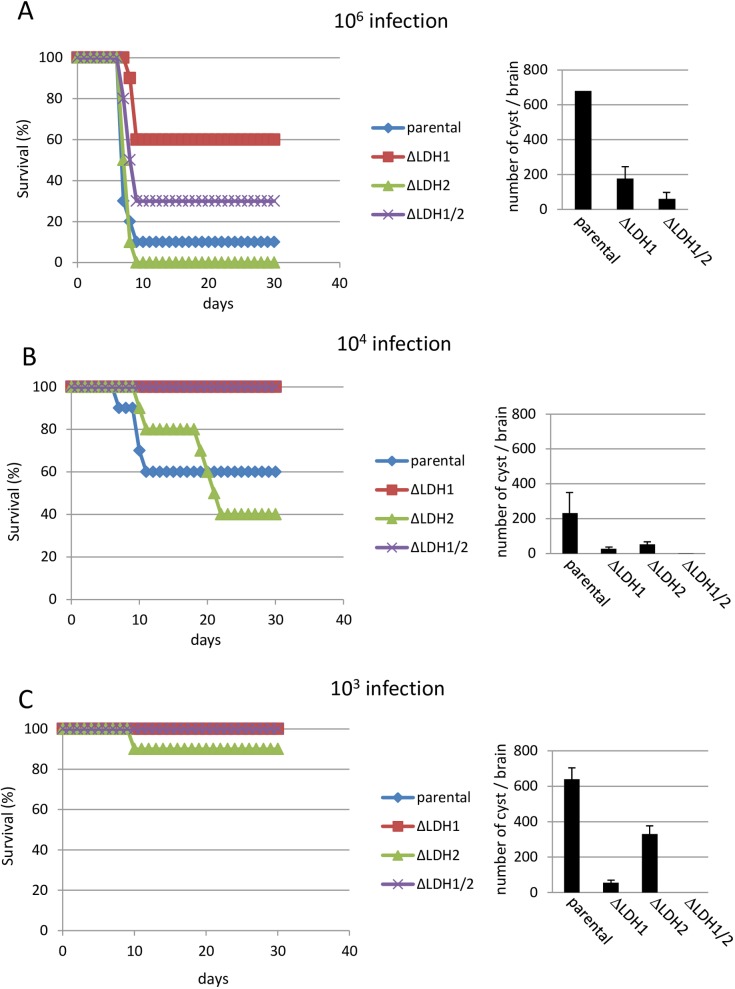
Virulence and cyst formation of LDH knockout strains in Balb/c mice. Left panels show survival of mice infected with indicated number of parasites (A: 10^6^, B: 10^4^, C: 10^3^). Right panels show the number of cyst in brains of survived mice.

To examine the effect of LDH knockout on parasite acute virulence, mouse infection studies were performed. Groups of Balb/c mice were infected with the indicated number of tachyzoites intraperitoneally and mortality was monitored daily for one month. After infection with 10^6^ tachyzoites, groups of parental or *Δldh2* strain infected mice demonstrated a 10 and 0% survival rate, respectively (Fig 4A, left). On the other hand, groups of mice challenged with *Δldh1* and *Δldh1Δldh2* strains demonstrated a 60 and 30% survival rate, respectively (Fig 4A, left). At a challenge dose of 10^4^ tachyzoites, groups of parental and *Δldh2* strain infected mice demonstrated a 60 and 40% survival rate, respectively. On the other hand, *Δldh1* and *Δldh1Δldh2* strain infected mice showed 100% survival (Fig 4B, left). Only the group of mice infected with *Δldh2* showed low (10%) mortality after low dose (10^3^) infection (Fig 4C, left). These results suggest that deletion of LDH1, but not LDH2, attenuated parasite virulence.

Cyst burdens in surviving mice were examined. Average number of cysts in the parental group was 680 (10^6^ infection, n = 1, Fig 4A, right), 231 ± 116 (10^4^, n = 6, Fig 4B, right) and 640 ± 72 (10^3^, n = 4, Fig 4C, right). Average number of cysts in the *Δldh1* group were 177 ± 62 (10^6^, n = 6, Fig 4A, right), 27 ± 7 (10^4^, n = 10, Fig 4B, right) and 56 ± 5 (10^3^, n = 5, Fig 4C, right). Average number of cysts in the *Δldh2* group was 52 ± 11 (10^4^, n = 4, Fig 4B, right) and 330 ± 34 (10^3^, n = 5, Fig 4C, right). Average number of cysts in the *Δldh1Δldh2* double knockout group was 60 ± 40 (10^6^, n = 3, Fig 4A, right), 1 ± 1 (10^4^, n = 10, Fig 4B, right) and 0 (10^3^, n = 5, Fig 4C, right). These results indicate that deletion of lactate dehydrogenase (*LDH1* or *LDH2*) reduced chronic stage cyst burdens in mice. The double knockout of *LDH1/LDH2* most severely impaired the development of cyst burdens.

### Protective immunity after primary infection

**Fig 5:**
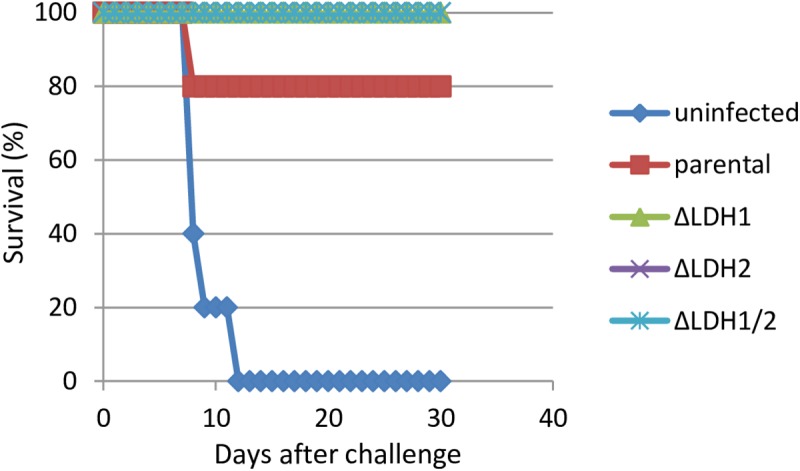
Protective immunity after primary infection. 10^3^ tachyzoites of LDH knockout and parental strains were infected into Balb/c mice. Mice were challenged with 100 parasites of virulent RH strain 30 days after primary infection and were monitored for another 30 days for survival. Group of naïve mice were also challenged (uninfected).

To examine protective immunity after primary infection, groups of Balb/c mice were vaccinated with a low-dose with 10^3^ tachyzoites of the parental or LDH knockout strains and thirty days later mice were challenged with 100 highly virulent (LD100) RH strain parasites. Group of non-vaccinated age-matched naive control mice were also challenged. As shown in Fig 5, all mice in the naïve group died within 11 days after challenge, one mouse died in parental strain infected group, and all mice survived in the other vaccinated groups. These results indicate that vaccination of mice with the LDH knockout strains elicited a fully protective immunity to virulent challenge infection.

## Discussion

The lactate dehydrogenase enzymes of *Toxoplasma gondii*, LDH1 and LDH2, catalyze the last committed enzyme step in the glycolytic pathway, interconversion of pyruvate and lactate. LDH activity is utilized for energy supply in anaerobic growth conditions [17, 18], however, the precise role of these LDH enzymes in virulence and cyst formation in *T*. *gondii* has not been conclusively determined. Understanding the potential role of LDH enzymes in the virulence and differentiation phenotypes of apicomplexan parasites could be important for the development of vaccines or therapeutic reagents.

LDH gene knockout parasites were produced and analyzed. These genetic deletion results show that *LDH1* and *LDH2* are not essential for growth of the acute tachyzoite stages *in vitro*. This was confirmed by demonstrating that all of these LDH knockout strains replicated as well as parental parasites during tachyzoite stage development. Since previous studies have reported that knockdown of LDH1 RNA expression [21] or inhibition of LDH enzyme activity by gossypol [20] resulted in a reduced parasite growth rate, our results suggest that these earlier methods targeting LDH RNA or protein may have had unintended off-site target effects that affected parasite growth. Our results indicate that LDH expression is dispensable for the parasite growth during the tachyzoite stages.

Remarkably, the cyst wall was not efficiently stained *in vitro* in the *Δldh1* or the *Δldh1Δldh2* strains after bradyzoite differentiation by *Dolichos biflorus* lectin staining. In contrast, no defect in cyst wall staining was observed in the *Δldh2* strain. These results strongly suggest that expression of LDH1 is required for differentiation of tachyzoites to bradyzoites and tissue cysts. The potential mechanism for LDH1 in differentiation is unclear at this time with respect to whether LDH1 plays a metabolic role in differentiation or a regulatory role, for example, similar to parasite enolase that has been shown to be a nuclear regulator of parasite gene transcription [29].

In addition to the specific defect of the *LDH1* knockout in bradyzoite differentiation *in vitro*, the *LDH1* knockout also demonstrated reduced virulence compared to parental or *LDH2* deleted strains. To understand the relationship between LDH1 and acute virulence, we examined motility, invasion efficiency and dissemination of *Δldh1* strain *in vitro*. However, the data obtained in these experiments did not reveal any significant differences between parental or knockout strains (data not shown).

The reduced virulence and the reduced ability of the *LDH1* knockout strain to differentiate from the tachyzoite to the bradyzoite stage correlated with markedly reduced cyst burdens and therefore reduced chronic infection *in vivo*. In contrast, while the *LDH2* knockout strain did not reveal any defects in acute virulence or in bradyzoite differentiation, the *Δldh2* strain, remarkably, also exhibited a significant defect in cyst burdens and the development of chronic infection *in vivo*. The mechanism for this defect in chronic infection is not known at this time, but could be associated with altered dissemination patterns, altered host response, cyst stability, or cyst maintenance. Further investigation is required to elucidate the specific defect of the *Δldh2* strain in cyst burdens. Nonetheless, the cyst burden defect in the *Δldh1* strain was greater than the defect observed in the *Δldh2* strain. Moreover, the double knockout *Δldh1/Δldh2* strain, exhibited the most markedly reduced cyst burdens in chronic infection. Previously, it has been reported that targeted deletion of glycolytic *T*. *gondii* enolase 1 also resulted in a reduction of brain cyst burden in chronically infected mice [29]. These results together with LDH deletion suggests that energy supply by glycolysis in the bradyzoite stages is likely to be important for developing and/or maintaining cyst burdens during chronic infection.

Recently, deletion of the orotidine 5′-monophosphate decarboxylase and uridine phosphorylase genes resulted in loss of replication and virulence in mice, and vaccination with this mutant strain elicited protective immunity [30]. All mice immunized with a low dose of LDH gene deletion mutants survived subsequent lethal challenge infection. These findings reveal that LDH knockout parasites can also confer protection immunity. Consequently, LDH knockout parasites, particularly the *Δldh1Δldh2* mutant, also represent potential vaccine strains owing to its ability to form little to no cysts, although complete inability of cyst formation is required for safe vaccine. In addition, lactate dehydrogenase in *T*. *gondii* controls virulence, bradyzoite differentiation, and chronic infection. Additional complementation studies are still necessary to conclusively verify that the phenotypes reported here for knockouts of LDH1 and/or LDH2 arise strictly from the absence of these glycolytic enzymes. Given the fact that LDH appears to be also important to other apicomplexan parasites, further investigation of LDH may uncover new roles for LDH in parasite differentiation and immunity.
